# Imaging manifestations of Caroli disease with autosomal recessive polycystic kidney disease: a case report and literature review

**DOI:** 10.1186/s12884-021-03768-8

**Published:** 2021-04-12

**Authors:** Xiuzhen Yao, Weiqun Ao, Jianhua Fang, Guoqun Mao, Chuanghua Chen, Lifang Yu, Huaijie Cai, Chenke Xu

**Affiliations:** 1 Department of Ultrasound, Shanghai Puotuo District People’s Hospital, Shanghai, China; 2 Department of Radiology, Tongde Hospital of Zhejiang Province, Hangzhou, Zhejiang China; 3 Department of Ultrasound, Affiliated Hangzhou First People’s Hospital, Zhejiang University School of Medicine, Hangzhou, Zhejiang China

**Keywords:** Caroli disease, Autosomal recessive polycystic kidney disease, Ultrasound, Magnetic resonance imaging, Diagnosis

## Abstract

**Background:**

Both Caroli disease (CD) and autosomal recessive polycystic kidney disease (ARPKD) are autosomal recessive disorders, which are more commonly found in infants and children, for whom surviving to adulthood is rare. Early diagnosis and intervention can improve the survival rate to some extent. This study adopted the case of a 26-year-old pregnant woman to explore the clinical and imaging manifestations and progress of CD concomitant with ARPKD to enable a better understanding of the disease.

**Case presentation:**

A 26-year-old pregnant woman was admitted to our hospital for more than 2 months following the discovery of pancytopenia and increased creatinine. Ultrasonography detected an enlarged left liver lobe, widened hepatic portal vein, splenomegaly, and dilated splenic vein. In addition, both kidneys were obviously enlarged and sonolucent areas of varying sizes were visible, but color Doppler flow imaging revealed no abnormal blood flow signals. The gestational age was approximately 25 weeks, which was consistent with the actual fetal age. Polyhydramnios was detected but no other abnormalities were identified. Magnetic resonance imaging revealed that the liver was plump, and polycystic liver disease was observed near the top of the diaphragm. The T1 and T2 weighted images were the low and high signals, respectively. The bile duct was slightly dilated; the portal vein was widened; and the spleen volume was enlarged. Moreover, the volume of both kidneys had increased to an abnormal shape, with multiple, long, roundish T1 and T2 abnormal signals being observed. Magnetic resonance cholangiopancreatography revealed that intrahepatic cystic lesions were connected with intrahepatic bile ducts. The patient underwent a genetic testing, the result showed she carried two heterozygous mutations in PKHD1. The patient was finally diagnosed with CD with concomitant ARPKD. The baby underwent a genetic test three months after birth, the result showed that the patient carried one heterozygous mutations in PKHD1, which indicated the baby was a PKHD1 carrier.

**Conclusions:**

This case demonstrates that imaging examinations are of great significance for the diagnosis and evaluation of CD with concomitant ARPKD.

## Background

Caroli disease (CD) is an extremely rare congenital disorder characterized by segmental and cystic dilation of the nonobstructive intrahepatic bile duct. In complex CD, cystic dilatation of the intrahepatic bile duct is often accompanied by liver fibrosis, cirrhosis, portal hypertension, and esophageal varices [1]. Autosomal recessive polycystic kidney disease (ARPKD) is a severe form of polycystic kidney disease with an estimated incidence of 1 in 20,000 [2]. Both CD and ARPKD are rare autosomal recessive disorders, and both are claimed to be related to mutations in polycystic kidney and hepatic disease 1 (PKHD1) [3] (i.e., the only pathogenic gene currently known). The two diseases are caused by the expression of the same gene mutation in different parts of the liver and kidney to differing degrees, and they are pathologically similar [4]. Clinical reports of CD with concomitant ARPKD are scant. It occurs more commonly in infants and children, for whom survival to adulthood is rare [5, 6]. CD with concomitant ARPKD commonly manifests as cystic dilation of the intrahepatic bile duct, portal hypertension, splenomegaly and enlarged kidneys with numerous cyst. Certain patients have symptoms such as fever, unexplained abdominal pain, and cholangitis, and lack characteristic symptoms, leading to misdiagnosis easily occurring, the failure of liver and kidney are generally present in the late stage of this disease and indicate a poor outcome [5–7]. Therefore, early diagnosis and intervention can improve the survival rate of patients to a certain extent. Imaging examinations represent the main method of diagnosis for the disease. This paper reports a 26-year-old pregnant woman with typical imaging manifestations, and genetic testing confirmed PKHD1 mutation. Through this rare case, we explored the clinical and imaging manifestations and progress of CD with concomitant ARPKD to improve the understanding of this disease.

## Case presentation

A 26-year-old pregnant woman with a gestational age of 25 + 2 weeks was admitted to hospital for more than 2 months after the discovery of pancytopenia and increased creatinine. She was admitted to the hospital for further diagnosis and treatment and categorized as “acute kidney injury, pancytopenia, and pregnancy” by the outpatient clinic.

The patient reported that her gums bled when she brushed her teeth; she had large, long-lasting bruises when she collided with hard objects; and she experienced no lower limb edema or backache. In terms of previous medical history, the patient was healthy, with no history of surgery, food or drug allergies, substance abuse, or exposure to toxins, dust, or harmful substances.

The patient did not undergo genetic testing before pregnancy, and the disease was not discovered until the second-trimester. Concerning menstrual history, the patient had normal menstrual volume, no dysmenorrhea, and a regular menstrual period. She had no fertility problems, had not experienced menopause, and currently had no children. Furthermore, her family had no history of malignant tumors.

The patient’s physical examination results were as follows. Her body temperature was 36.5 °C, pulse was 76 beats/min, respiration was 20 breaths/min, and blood pressure was 140/88 mmHg. She was conscious with satisfactory mental wellbeing. She had a centered trachea and clear respiratory sounds with no abnormal sounds in either lung. Her heart rate was normal with no abnormal sounds. Abdominal distension; consistent with gestational age; no tenderness; rebound tenderness. Her liver was not palpable under the ribs, but her spleen was palpable 1 cm under the ribs and hard with no tenderness. No tenderness or percussion pain were noted in either kidney, and no tenderness was noted in either ureter.

The patient’s laboratory examination results were as follows: white blood cells: 2.32*10^9^/L; hemoglobin: 65 g/L; red blood cells: 2.65*10^12^/L; platelets: 31*10^9^/L; serum ferritin: 7.1 μg/L; and creatinine 246 μmol/L. CA19–9, CA-125 alpha-fetoprotein, and carcinoembryonic antigen were in the normal range. Blood electrolytes were normal. A bone marrow examination was performed on July 17, 2020, and the results were as follows. The bone marrow morphological description revealed active nucleated cell proliferation; active granulocyte hyperplasia, mainly in the myelocyte and metamyelocyte stages; active erythrocyte hyperplasia, mainly in the immediate and late erythroblast stages; and reduced nucleoplasm in some early erythroblasts. Her bone marrow morphology revealed iron-deficiency anemia.

**Fig. 1:**
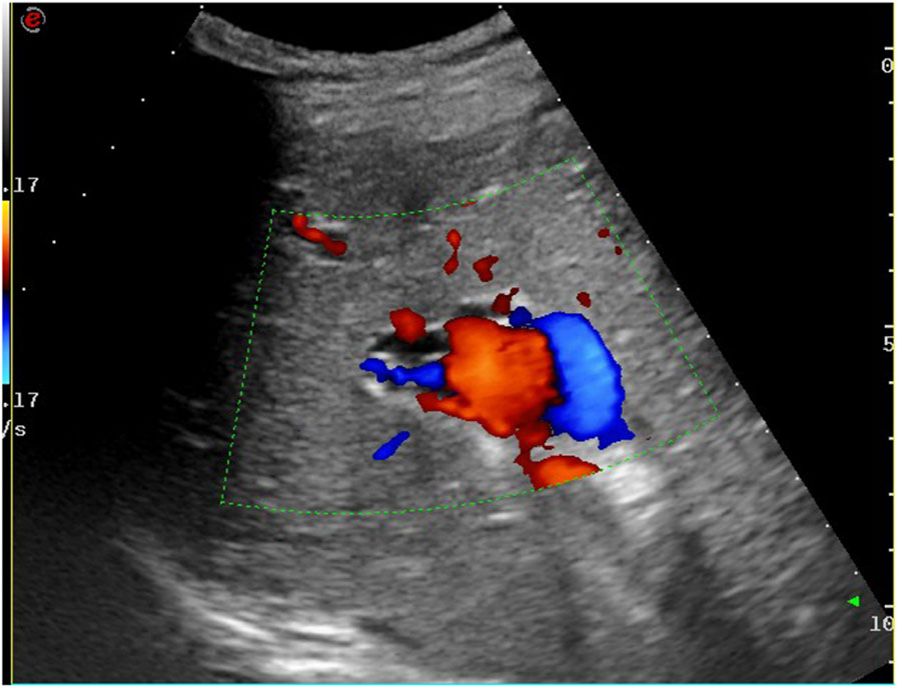
Reduced echo in the right liver lobe, thickened bright spot, tortuously dilated portal vein, and color flow signal displays in color Doppler flow imaging, accompanied by slightly dilated bile ducts

**Fig. 2:**
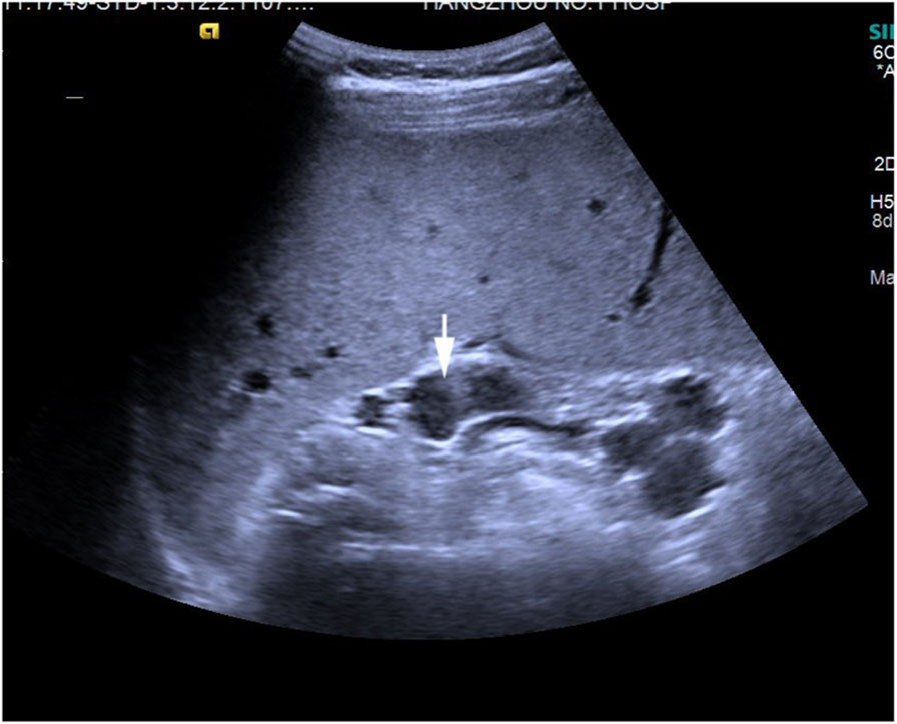
Enlarged spleen volume, full shape, slightly enhanced intradermal echo, fine bright spot, spleen thickness of 5.7 cm, tortuously dilated splenic vein at the hilum (arrow), and the wider part is approximately 1.51 cm

**Fig. 3:**
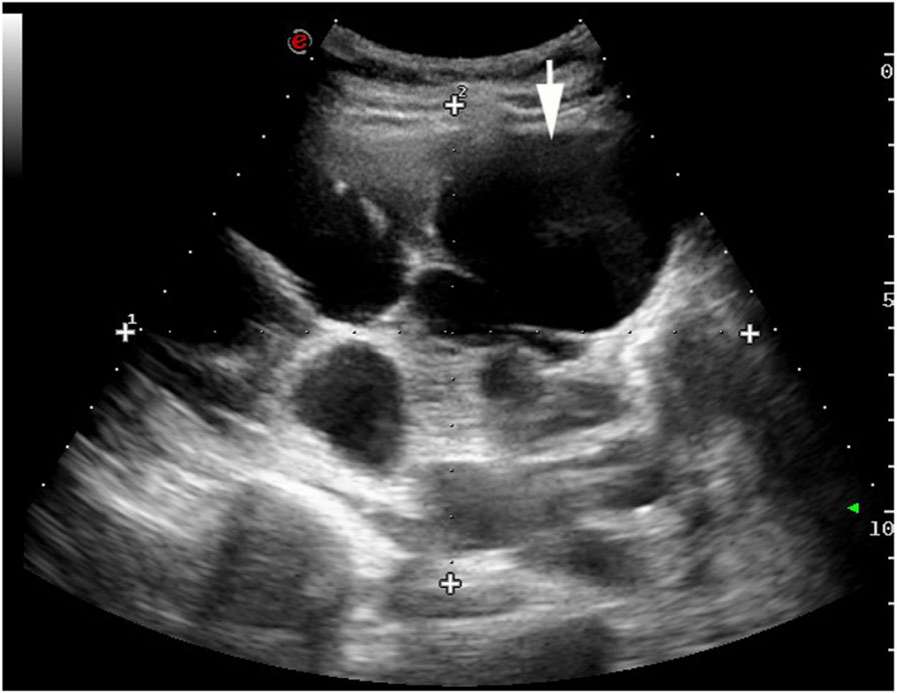
Both kidneys were enlarged in size and abnormal in shape. Normal renal parenchyma was almost unobservable. The renal cortex was thin, and numerous anechoic, honeycomb-shaped dark areas of varying sizes could be observed (arrow)

The patient underwent ultrasonography, magnetic resonance imaging (MRI), and magnetic resonance cholangiopancreatography (MRCP) examinations. Ultrasonic examination revealed an enlarged left liver lobe and a moderately sized and abnormally shaped right liver lobe. The width of the main vein of the porta hepatis was approximately 1.7 cm with local tortuous dilatation in a “vermis” shape (Fig. 1). Color Doppler flow imaging (CDFI) indicated that the main portal vein was the signal of blood flowing into the liver, and its peak flow velocity measured by pulse-Doppler was approximately 35.8 cm/s; the accompanying dilated vein near the portal trunk was the signal of hepatic outflow, and its peak flow velocity measured by pulse-Doppler was approximately 21.4 cm. The spleen was approximately 5.3 cm thick; it was plump with a smooth outline, a strong echo in essence, and a dilated splenic vein, which was approximately 1.5 cm wide at the hilum of the spleen (Fig. 2). Both kidneys were obviously enlarged, abnormally shaped, and covered with anechoic dark areas of different sizes (Fig. 3); no abnormal blood flow signal was detected by CDFI.

Regarding the fetus, the gestational age was approximately 25 weeks, which was consistent with the actual fetal age. No significant abnormalities were observed except for polyhydramnios. The limb structure and face of the fetus were normal. In addition, no abnormalities were observed in the fetal spine, intracranial pressure, heart, lungs, kidneys, gastric vesicles, intestines, gallbladder, or bladder. The amniotic fluid index was 24–25 cm (reference value is 8–18 cm) with a maximum depth was 10.2 cm.

**Fig. 4:**
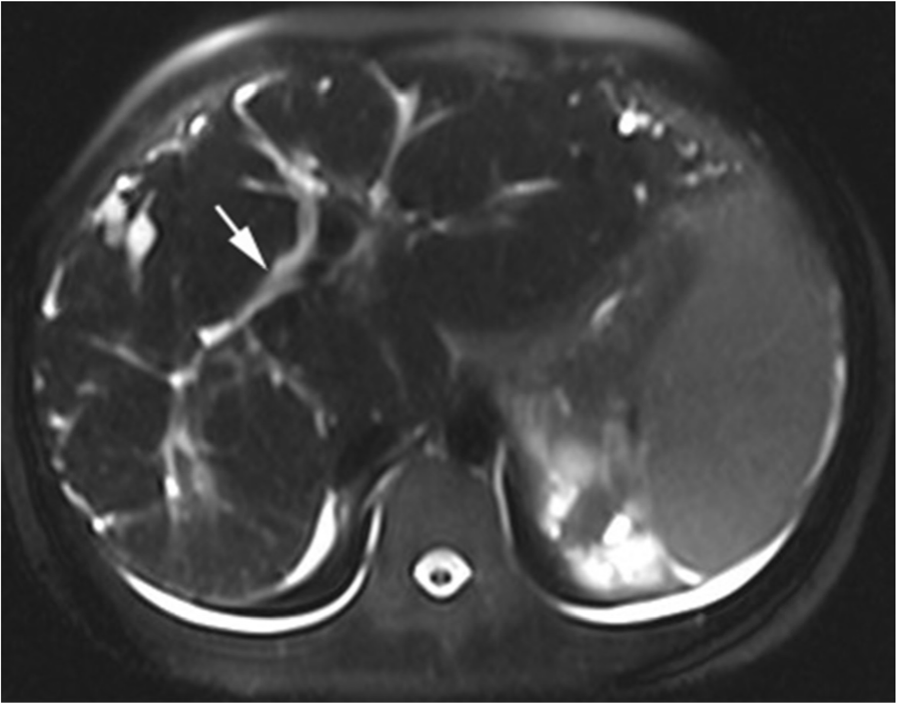
Hepatomegaly and splenomegaly could be observed in the MRI-T2WI; a high cystic signal was identified on the liver surface; and no dilation was observed in the common bile duct (arrow)

**Fig. 5:**
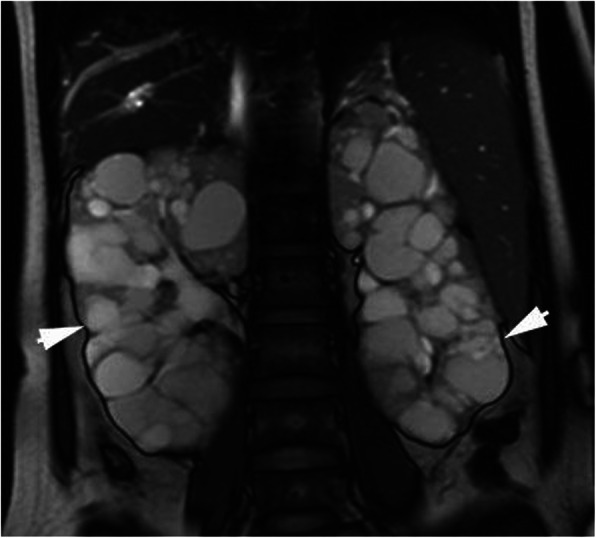
Both kidneys increased diffusely in the MRI-T2WI; normal structures disappeared with scattered high polycystic T2WI signals of different sizes (arrow)

**Fig. 6:**
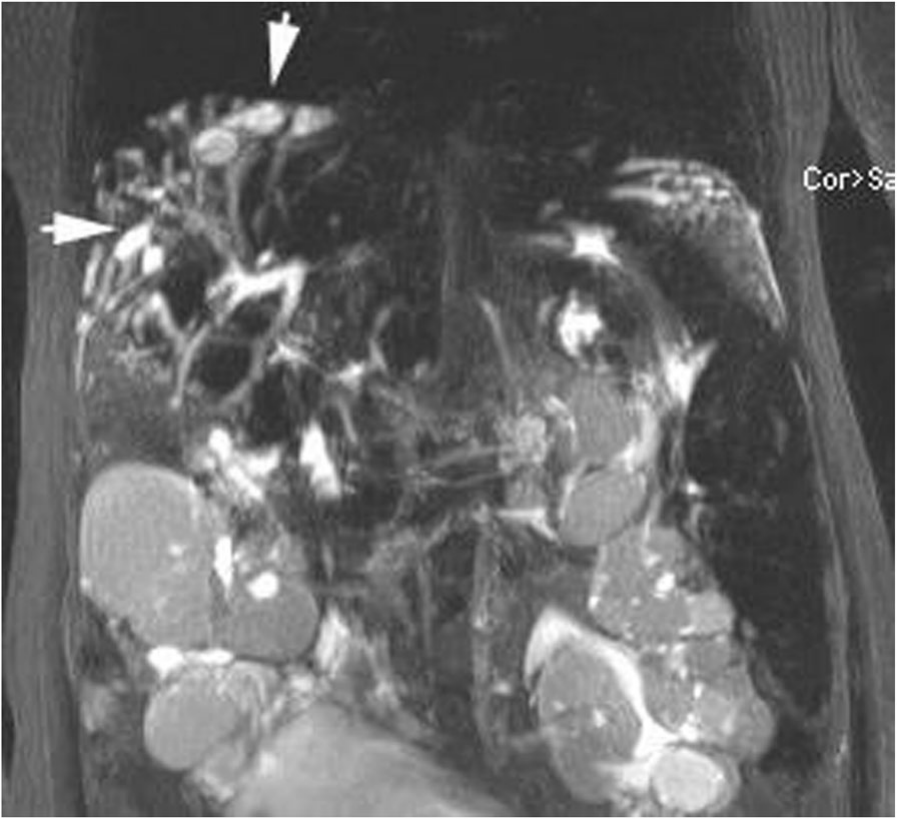
MPCP revealed a high polycystic signal (arrow) at the distal intrahepatic bile duct, which was connected to the intrahepatic bile duct, and said duct was slightly dilated

Maternal MRI revealed that the liver was plump with disproportionate liver lobes. Furthermore, it revealed hepatomegaly as well as multiple tortuous dilated bile duct shadows near the liver surface, among which polycystic liver disease was identified near the top of the diaphragm. The T1 weighted image (WI) was the low signal, the T2WI was the high signal, and no dilation was observed in the common bile duct (Fig. 4); furthermore, the portal vein was widened and the spleen volume was enlarged. The volume of the kidneys had increased to an abnormal shape, with multiple, long, roundish T1 and T2 abnormal signals were observed (Fig. 5). MRCP revealed that the intrahepatic cystic lesions were connected to the intrahepatic bile ducts (Fig. 6). In conclusion, CD, polycystic kidney disease, cirrhosis, portal hypertension, and splenomegaly were revealed.

The patient’s venous blood samples were taken in July 2020 for genetic testing (whole exome sequencing, WES). The results revealed that the patient carried two heterozygous mutations in PKHD1: c.2854G > A, causing a change in amino acid P.(Gly952Arg), and in c.4682G > A, causing a change in amino acid P.(Cys1561Tyr).

According to the imaging examinations and genetic testing, the patient was finally diagnosed with CD with concomitant ARPKD. In this state, the ongoing pregnancy would lead to higher risks for both the mother and fetus. Iron sucrose was infused to correct the iron-deficiency anemia. In addition, methylprednisolone succinate (20 mg) was prescribed to increase platelets, supplemented by stomach protection and calcium supplement supportive treatments. Cesarean section was performed after 36 weeks of pregnancy, the baby is normal after examination. Three months later in October 2020, the baby underwent a genetic test (WES), the result showed that the patient carried one heterozygous mutations in PKHD1: c.2854G > A, causing a change in amino acid P.(Gly952Arg), which indicated the baby was a PKHD1 carrier.

## Discussion and conclusions

This case report presented a 26-year-old pregnant woman with her liver and kidneys both affected. She had anemia and renal insufficiency, and ultrasound confirmed that the fetal organs were normal. The baby underwent a genetic test at 3 months old, the result confirmed that the baby was a PKHD1 carrier. Relevant studies [5–7] have reported that CD with concomitant ARPKD often presents as a urinary tract infection accompanied by renal insufficiency. CD with concomitant ARPKD is characterized by hepatomegaly with congenital liver fibrosis, nonobstructive cystic dilatation of the intrahepatic bile duct, and abnormal ductal plate development. Further progress of this disease can cause the patient to have portal hypertension and open collateral circulation, which was represented by the liver cirrhosis and portal hypertension in the image [7]. The clinical manifestations of liver disease were insidious. Elevated liver transaminase can cause hypoalbuminemia and reduction in coagulation factors. In addition, retrograde cholangitis and portal hypertension are commonly seen. Gastrointestinal bleeding caused by portal hypertension complications is a risk factor that can lead to death [8]. ARPKD is a medullary cystic kidney disease. In severe cases, the renal cortex and medulla will be affected, eventually leading to hypertension and chronic renal insufficiency. ARPKD most commonly occurs in the fetal stage and may be accompanied by pulmonary dysplasia. Approximately 30–40% fetuses die in the perinatal period, and nearly 25% of survivors develop end-stage renal disease before the age of 11 years and require kidney transplantation [9].

Imaging examinations are the primary method for diagnosing CD with concomitant ARPKD, with genetic testing being an essential supplement. This case was confirmed by genetic testing to be a mutation of PKHD1, and a relevant study reported that PKHD1 may be a major gene of CD [10]. Variants in PKHD1 are responsible for ARPKD and CD with high inter- and intrafamilial phenotypic variability. This disease is an autosomal recessive disorder, for which more than 300 types of mutation have been reported to date, which are mainly missense mutations, with a few leading to protein-truncating variants [11]. Certain scholars have used abdominal ultrasound, MRI, and PKHD1 gene mutation detection as effective means for the early diagnosis of ARPKD [12]. Abdominal imaging is the most commonly used method for clinical diagnosis of the disease. The use of high-resolution ultrasound, computed tomography scans, and MRI can improve diagnosis sensitivity. Ultrasound can be used to observe the dilation progress of the patient’s intrahepatic bile duct multiple times in real time without invasiveness and radiation. Moreover, it can observe portal hypertension, changes in spleen size, and the venous vessel diameter and vascular flow rate. Because of the particularity of this patient being a pregnant woman, ultrasound demonstrated advantages for dynamically observing changes of the fetus in real time, and fetal ultrasound is a preferred imaging technique. During the follow-up process, liver stiffness was measured by ultrasound elastography. Contrast-enhanced ultrasound can observe changes of capillaries in the liver or kidney, and can also evaluate changes in liver and kidney function [13, 14]. Moreover, the patient had dilated intrahepatic bile ducts without severe obstruction; thus, close follow-up observation was selected. If further obstruction and dilatation of the internal bile ducts were identified, biliary drainage could have been performed under the guidance of ultrasound. Fully utilizing ultrasonography for this rare disease can provide numerous valuable information, greatly increasing clinicians’ confidence in diagnosis and treatment. MRI provides superior soft tissue resolution, in which T2WIs can clearly reveal liver and kidney cystic lesions and objectively evaluate the degree of polycystic liver, cirrhosis, liver fibrosis, and polycystic kidney. In addition, MRCP can evaluate the severity of disease and clearly illustrate the pathological changes in intrahepatic bile ducts.

Through imaging, CD commonly manifests as cystic dilation of the intrahepatic bile duct, usually containing stones, and exhibiting a central dot sign in MRI. Said sign corresponds to a portal-vein radicle and an accompanying hepatic artery branch protruding into the lumen of the dilated bile duct. MRCP can clearly present the pathological changes in intrahepatic bile ducts and their dilatation. Secondary biliary cirrhosis accompanied by portal hypertension and splenomegaly often occur in the liver. ARPKD manifests as increased kidney volume with polycystic echoes or signals in the parenchyma [15].

To our knowledge, Only six cases of pregnant women with CD/ARPKD were reported in the literature [5, 16, 17] (Table 1). Other literature reported that the father was the CD/ARPKD patient or the carrier, causing fetal illness or miscarriage of pregnant women [18]. Our case was a young pregnant woman with CD and concomitant ARPKD, but the fetus was developing normally, which was exceptionally rare in clinical practice. In view of the independent occurrence and development of liver and kidney disease, close long-term follow-up is required once the diagnosis is confirmed. Therefore, this study recommends rechecking the liver and kidney imaging, routine complete blood count, and liver and kidney function. Imaging can accurately grasp the occurrence, development, and surgical treatment of portal hypertension complications to prevent gastrointestinal bleeding. In terms of the kidneys, hypertension, renal insufficiency, and accompanying urinary tract infection are likely to occur as the disease progresses. Therefore, infections must be actively controlled during follow-up. If bile duct obstruction occurs, biliary drainage can be administered under the guidance of ultrasound.

**Table 1:** Clinical and Imaging characteristics in pregnant women with CD/ARPKD

Ref.	Age	Clinical manifestation	Outcomes	Imaging fingings
Banks N [5]	21	A transient impairment of renal function	VD, LB	None
	23	None	VD, LB	Endoscopy: mild esophageal varices MRI: liver fibrosis and portal hypertension, enlarged kidneys with multiple cysts
	23	Right upper quadrant pain	VD, LB	None
	33	None	CS, LB. Received both kidney and liver transplantation at age 59	None
Salati SA [16]	25	Chronic renal failure	VD, LB	Ultrasound (fetus): enlarged kidneys with multiple cysts
Tsunoda M [17]	19	None	VD, LB	MRI: numerous cysts in the liver and polycystic kidney disease
Our case	26	Acute kidney injury, pancytopenia	CS, LB	Ultrasound: enlarged liver lobe, widened hepatic portal vein, splenomegaly, both enlarged kidneys with multiple cysts and no abnormal blood flow signals MRI: polycystic liver disease and enlarged kidneys with multiple cysts MRCP: intrahepatic cystic lesions were connected to the intrahepatic bile ducts

Note: VD: vaginal delivery; LB: livebirth; CS: cesarean section

In summary, this study reported a rare case of CD with concomitant ARPKD in a young pregnant woman. Imaging examination plays a crucial role in the diagnosis and follow-up review of the disease.

## Data Availability

The datasets used and/or analyzed during the current study are available from the corresponding author on reasonable request.

## Abbreviations

ARPKD: autosomal recessive polycystic kidney disease
CD: Caroli disease
CDFI: Color Doppler flow imaging
CS: Cesarean section
LB: Livebirth
PKHD1: Polycystic kidney and hepatic disease 1
MRI: Magnetic resonance imaging
MRCP: Magnetic resonance cholangiopancreatography
VD: Vaginal delivery
WES: Whole exome sequencing
WI: Weighted image
